# Experimental Life History Evolution Results in Sex-specific Evolution of Gene Expression in Seed Beetles

**DOI:** 10.1093/gbe/evac177

**Published:** 2022-12-21

**Authors:** Elina Immonen, Ahmed Sayadi, Biljana Stojković, Uroš Savković, Mirko Đorđević, Johanna Liljestrand-Rönn, R Axel W Wiberg, Göran Arnqvist

**Affiliations:** Evolutionary Biology, Department of Ecology and Genetics, Uppsala University, Uppsala, Sweden; Department of Medical Sciences, Uppsala University, Uppsala, Sweden; Department of Evolutionary Biology, Institute for Biological Research “Siniša Stanković”, National Institute of the Republic of Serbia, University of Belgrade, Belgrade, Serbia; Faculty of Biology, Institute of Zoology, University of Belgrade, Belgrade, Serbia; Department of Evolutionary Biology, Institute for Biological Research “Siniša Stanković”, National Institute of the Republic of Serbia, University of Belgrade, Belgrade, Serbia; Department of Evolutionary Biology, Institute for Biological Research “Siniša Stanković”, National Institute of the Republic of Serbia, University of Belgrade, Belgrade, Serbia; Animal Ecology, Department of Ecology and Genetics, Uppsala University, Uppsala, Sweden; Evolutionary Biology, Department of Ecology and Genetics, Uppsala University, Uppsala, Sweden; Animal Ecology, Department of Ecology and Genetics, Uppsala University, Uppsala, Sweden

**Keywords:** longevity, experimental evolution, RNA-seq, aging, life history evolution, sex bias

## Abstract

The patterns of reproductive timing and senescence vary within and across species owing to differences in reproductive strategies, but our understanding of the molecular underpinnings of such variation is incomplete. This is perhaps particularly true for sex differences. We investigated the evolution of sex-specific gene expression associated with life history divergence in replicated populations of the seed beetle *Acanthoscelides obtectus*, experimentally evolving under (E)arly or (L)ate life reproduction for >200 generations which has resulted in strongly divergent life histories. We detected 1,646 genes that were differentially expressed in E and L lines, consistent with a highly polygenic basis of life history evolution. Only 30% of differentially expressed genes were similarly affected in males and females. The evolution of long life was associated with significantly reduced sex differences in expression, especially in non-reproductive tissues. The expression differences were overall more pronounced in females, in accordance with their greater phenotypic divergence in lifespan. Functional enrichment analysis revealed differences between E and L beetles in gene categories previously implicated in aging, such as mitochondrial function and defense response. The results show that divergent life history evolution can be associated with profound changes in gene expression that alter the transcriptome in a sex-specific way, highlighting the importance of understanding the mechanisms of aging in each sex.

**Significance**

How an organism’s age is affected by their evolved life history schedules. Here, we utilize over 200 generations of experimental evolution in seed beetles to investigate the impact of life history evolution on gene expression. We show that adaptation to late-life reproduction that leads to extended lifespan is associated with reduced sex differences in gene expression, compared with faster life history where reproduction is restricted to early life. Transcriptomic changes are more numerous in females, aligned with their greater divergence in lifespan. Our results suggest that life history evolution is an important driver of sexually dimorphic transcriptome and implicate immunity and mitochondrial function as mechanisms involved in sex-specific aging.

## Introduction

Aging evolves as a side effect due to selection failing to maintain reproductive fitness at older ages (Charlesworth 1993, 2000; Hughes and Reynolds 2005). A large body of evidence shows that lifespan is subject to trade-offs with other fitness components, such as fecundity (Wilkinson and South 2002; Wang et al. 2013; Lemaître et al. 2015), but it can evolve and become extended when selection acts on associated life history traits (McHugh and Burke 2022). Several evolutionary studies have shown that selection for high resource allocation to growth or reproduction early in life is associated with earlier and/or more rapid aging (Flatt 2011; Flatt and Heyland 2011; McHugh and Burke 2022). Conversely, selection for delayed age of first reproduction typically increases somatic maintenance, late-life fecundity and longevity (Rose and Charlesworth 1981; Luckinbill et al. 1984; Rose 1984; Partridge and Fowler 1992; Tucić et al. 1996, 1997, 1998; Miyatake 1997; Partridge et al. 1999; Remolina et al. 2012; Carnes et al. 2015; McHugh and Burke 2022). However, males and females often differ in optimal reproductive strategies, selecting for different life histories (Vinogradov 1998; Maklakov and Lummaa 2013; Bonduriansky 2014; Hämäläinen et al. 2018). In most species, females outlive males (Promislow 1992, 2003; Liker and Székely 2005; Weadick and Sommer 2016). Sex differences in lifespan evolution is at least to some extent affected by the asymmetric inheritance of sex chromosomes (Xirocostas et al. 2020), as well as mitochondria. Due to maternal inheritance, mitochondrial genes are subject to selection only in females and therefore accumulation of deleterious mutations may increase mortality in males. Reduced male fitness subsequently selects for compensatory mutations on nuclear genes interacting with the mitochondria (Frank and Hurst 1996; Gemmell et al. 2004; Tower 2006; Camus et al. 2012; Maklakov and Lummaa 2013). Despite the fact that females and males typically differ in lifespan and associated life histories, the genetic architecture underlying the evolution of sex differences is still poorly characterized.

Sex differences in longevity and associated life history traits are inherently constrained if alleles in the shared loci with similar phenotypic effects in the sexes are selected in the opposite directions in females and males (Bonduriansky 2014; Immonen et al. 2018). The intersexual genetic correlation can be high and restrict independent evolution of lifespan in the sexes (Berg and Maklakov 2012), but the sexes can also show a considerable difference in the additive genetic basis of longevity variation (Lehtovaara et al. 2013). Phenotypic sex differences are largely achieved through modification of expression of shared genes. This predicts that life history evolution should affect sex bias in gene expression (Tower 2017). Males and females typically show vast gene expression differences (Griffin et al. 2013; Sharma et al. 2014; Lipinska et al. 2015; Dean and Mank 2016; Mayne et al. 2016; Shen et al. 2017; Darolti et al. 2018; Dutoit et al. 2018; Lichilín et al. 2021; Toubiana et al. 2021), which should be the consequence of both natural and sexual selection pressures but have largely been studied in the context of sexual competition (Mank 2017). Given that sex-specific selection on life history strategies is common and can affect multiple genetically correlated traits (Wedell et al. 2006; Immonen et al. 2018), evolution of traits associated with lifespan could influence genome-wide sex specificity of the transcriptome.

**Fig. 1.**
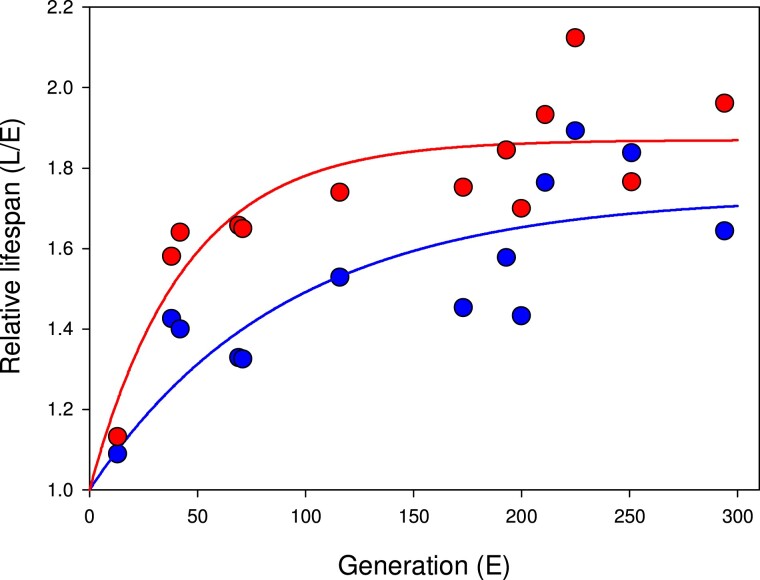
The unfolding of sex-specific (females: red; males: blue) divergence in lifespan between the E and L lines over time (mated individuals). Solid lines represent the fitted function *Y* = 1 + *a*(1 − *b^X^*). Female lifespan evolved more rapidly (*b*: *t* = 2.46, *P* = 0.030) and has reached a greater divergence (*a*: *t* = 2.83, *P* = 0.015) relative to male lifespan divergence. Adult life span was assayed by allowing newly enclosed males and females to mate, and then separating the sexes into petri dishes where life span was monitored by daily spot checks (see Tucić 1996, 1998 for details).

Here, we test how divergence in lifespan and life history has affected gene expression evolution of female and male *Acanthoscelides obtectus* seed beetles, using replicated long-term experimental evolution (EE) allowing for reproduction early or late in life. We focus on investigating expression divergence early in adult life, when selection favoring different resource allocation strategies has been strongest. Our EE lines were founded in 1986 and they consist of populations maintained for >200 generations under two contrasting life history regimes: enforced reproduction (E)arly in life (days 1–2 upon adult hatching) or only (L)ate in adult life (after day 10). Both EE regimes were replicated four times (Tucić et al. 1996, 1998). Previous studies have shown that life history adaptation to these regimes has been dramatic and predictable: compared with beetles from the E lines, the L beetles show decelerated senescence and live for significantly longer (more than twice as long; Đorđević et al. 2017), are larger (Šešlija et al. 2009), show a lower metabolic rate (Arnqvist et al. 2017) and distinct physiologies (Lazarević et al. 2012) as well as marked differences in the mitochondrial electron transport chain (ETC) complex activities (Đorđević et al. 2017). Interestingly, phenotypic evolution has been sex specific despite a shared EE life history regime, with generally more pronounced lifespan divergence in females than in males (fig. 1). For this study, we first sequenced, de novo assembled and annotated the *A. obtectus* genome, which we used for assembling the transcriptomes for the E and L lines. We then compared differential gene expression between different sexes and tissues using the replicate lines as biological replicates.

## Results

### Reference Genome Assembly

The genome assembly yielded 1.1 Gb in total size, containing 6,654 contigs with an N50 of 791 kb. Genome completeness assessment with BUSCO showed a very high fraction of well-assembled genes with 98% of the complete BUSCO genes detected in the assembly (supplementary Table S1, Supplementary Material online in the supplementary material and methods). The annotation pipeline identified 38,104 protein coding genes. The details of the annotation are presented in the supplementary Table S2 (Supplementary materials and methods, Supplementary Material online).

### Transcriptome Assembly and Annotation

The assembled transcriptome included 50,481 transcripts, corresponding to 32,006 genes. The GC content was 39.63%, which is consistent with other beetle genomes (i.e., *Dendroctonus ponderosae* at 36%; Keeling et al. 2013), and *Tribolium castaneum* at 33% (Tribolium Genome Sequencing Consortium 2008). The assembly's N50 length was 4,117 bases and a mean transcript size of 2,089.6 bases. Transcript length ranged from 200 bases to 50,358 bases, with 26,681 transcripts being >1 kb and 1,026 being >10 kb. 77% of all genes were represented as single isoforms. In addition to the primary statistics, we assessed the quality of the transcriptome assembly using the BUSCO tool, and detected 945 (97%) complete BUSCO list genes in our transcriptome.

### Gene Expression Divergence Under Early and Late Life History Regimes

**Fig. 2.**
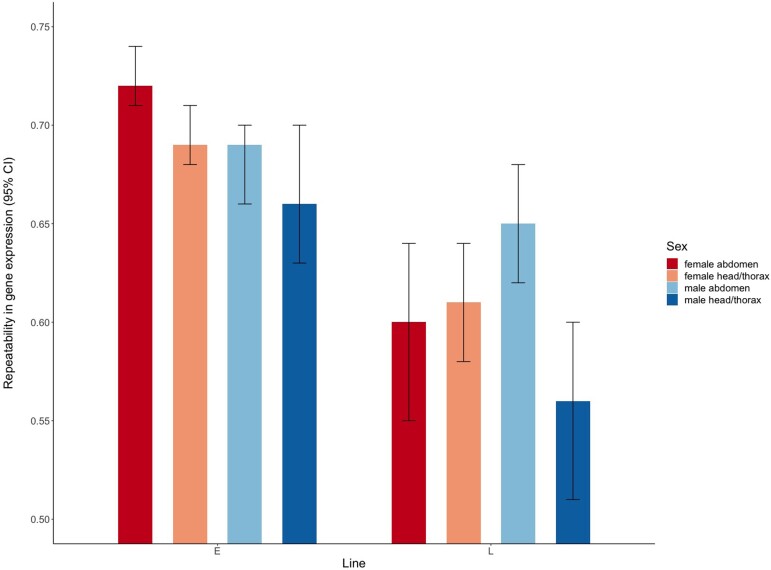
Repeatability estimates (with 95% bootstrap CI) of gene expression across the four replicate E and the four replicate L lines, for each sex and tissue.

**Fig. 3.**
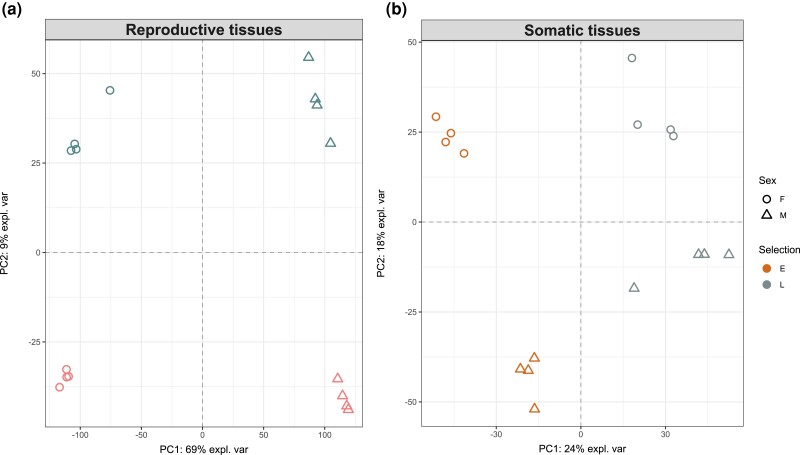
Principle component analysis of gene expression in the (*a*) reproductive and (*b*) somatic tissues of the E and L lines. The axes show the first and second principle components with the % of variance explained by each.

**Fig. 4.**
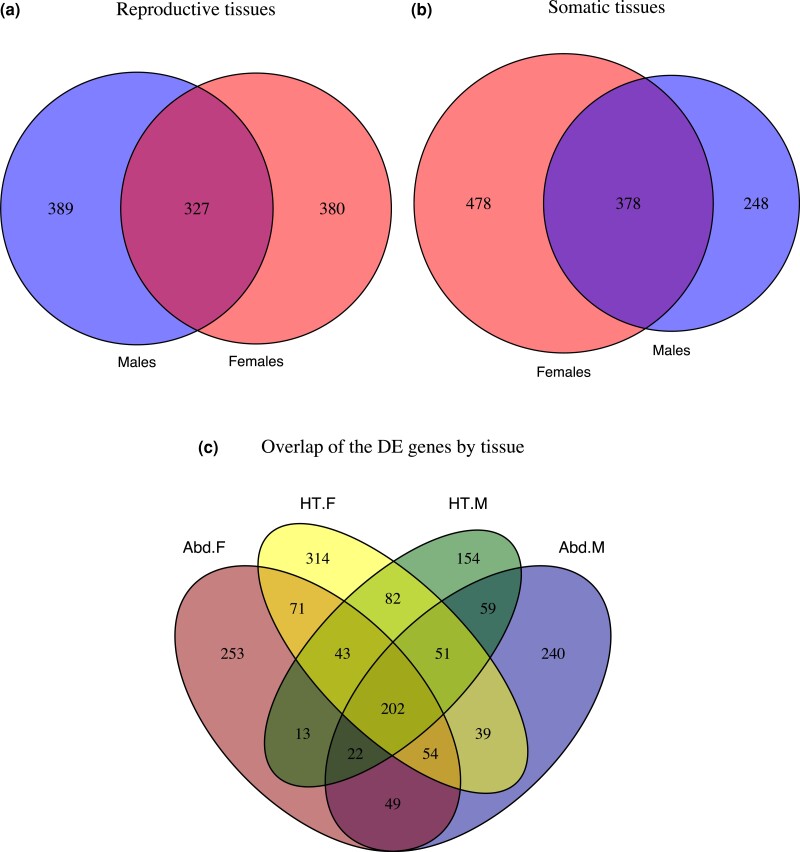
(*a* and *b*) Numbers of DE genes (*q* < 0.05, abs. logFC > 1) between the (*E*)arly and (*L*)ate EE regimes in each sex. (*c*) Overlap of genes significant in each tissue category and sex (Abd = abdomen; HT = head and thorax; F = females; M = males).

The multivariate repeatability of gene expression was generally high and varied between 0.56 and 0.72 across tissues, sexes, and EE regimes. Expression profiles were more repeatable in E lines than in L lines (fig. 2). As expected, sexual dimorphism in gene expression was more pronounced in the abdomen (dominated by reproductive tissues) than in the head and thorax (composed of somatic tissue; fig. 3). We identified a total of 1,646 differentially expressed (DE) genes between the E and L regimes across tissues and sexes (with *q*-value < 0.05 and abs. logFC > 1; supplementary Table S3, Supplementary Material online) revealing that a highly polygenic modulation of gene expression is responsible for the marked divergence in life histories seen between the E and L lines. About 1,096 of these genes were significant (in either sex) in the reproductive tissues and 1,104 in the somatic tissues (6.4% and 6.5% of the expressed genes analyzed, respectively; fig. 4*a*and*b*). Yet, when comparing the overlap of DE genes between the two tissue categories (fig. 4*c*), we detected only 202 genes that were similarly DE in both reproductive and somatic tissues in both sexes.

Overall, 70% of the DE genes showed some sex specificity. In the reproductive tissues of the abdomen, less than one-third were similarly DE in both sexes (327 genes), whereas 380 and 389 genes were significant only in females or males, respectively (fig. 4*a*). When considering DE genes detected exclusively in the abdomen (542 genes) the sex differences remained: only 9% (49 genes) were significant in both sexes, whereas 253 and 240 genes were significant only in females or males, respectively (fig. 4*c*). Ninety-three genes showed a more extreme sex-specific divergence, evident as a significant sex-by-selection interaction in expression (supplementary Table S3, Supplementary Material online). In the somatic tissues, nearly twice as many genes were DE between the E and L regimes in females compared with males (478 and 248 genes, respectively), whereas 378 genes were similarly DE in the sexes (fig. 4*b*). The pattern holds also when considering genes significant only in the somatic tissues (550 genes of which 314 and 154 genes were significant only in females or males, respectively, and 82 genes in both (fig. 4*c*). Seventy-seven genes showed a significant sex-by-selection interaction effect (supplementary Table S3, Supplementary Material online). In summary, the sexes largely differ in the genes showing greatest expression divergence. Although similar numbers of genes differ in expression in the reproductive tissues of males and females, in the somatic tissues the differences are more numerous in the females.

### Evolution of Sex-biased Expression

**Fig. 5.**
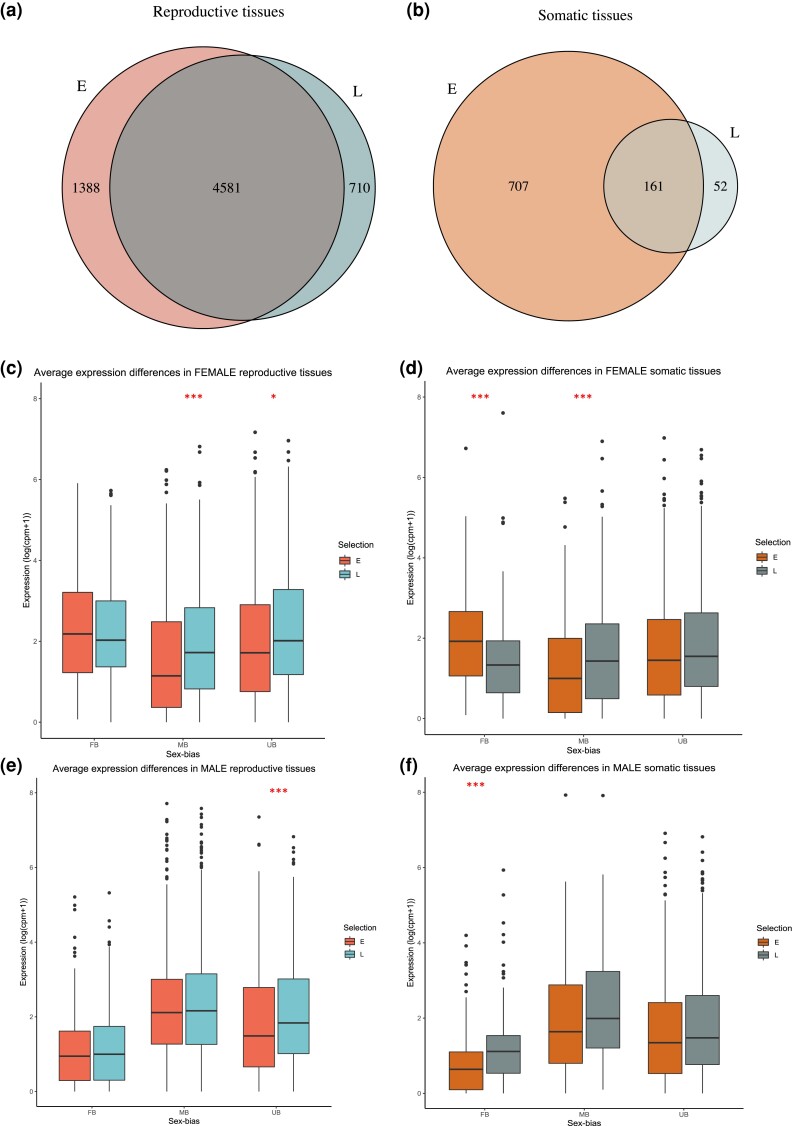
(*a* and *b*) Numbers of sex-biased genes in the E and L regimes. (*c*–*f*) Average expression of DE female-, male-, and un-biased genes in each sex. Significant median difference between the regimes: ***P < 0.001, *P < 0.05.

Given the implications of sex differences in how the E and L beetles have diverged, we next tested directly whether selection has impacted sexual dimorphism in expression by comparing sex-biased genes in each life history EE regime, within each tissue category. The transcriptomes of the E lines were more sexually dimorphic than of the L lines, in both reproductive and somatic tissue groups (fig. 5*a*and*b*). In the reproductive tissues, 35% (5,969) and 31% (5,291) of the analyzed genes were sex biased in the E and L lines, respectively. The E lines showed a significantly greater proportion of female-biased genes compared with the L regime (E: 2,922 genes, 17.1%; L: 2,504 genes, 14.7%; proportion difference *χ*^2^_1_ = 38.1, *P* < 0.0001). The same was true for the male-biased genes (E: 3,047 genes, 17.8%; L: 2,787 genes, 16.3%; proportion difference *χ*^2^_1_ = 13.9, *P* = 0.0002). The differences were even more pronounced in the somatic tissues, where we found four times more sex-biased genes in the E compared with the L regime (E: 868 genes, 5.0%; L: 213 genes, 1.2%, respectively). The proportion differences of female- and male-biased genes in the E compared with the L transcriptomes were significant (female-biased genes E: 546, 3.2%; L: 84, 0.49%; χ^2^_1_ = 343.7, *P* < 0.0001, *male-biased genes* E: 322, 1.9%; L: 129, 0.76%; *χ*^2^_1_ = 82.8, *P* < 0.0001).

We then tested whether sex-biased genes (of either E or L beetles) had disproportionally diverged in expression between the regimes, compared with un-biased genes. Here, we defined sex-biased genes as those with significant sex bias in either E or L regime (or in both), to compare with genes that lacked evidence of sex bias in both regimes. In the reproductive tissues, there was no evidence to support that expression divergence would have particularly affected sex-biased genes, and if anything, male-biased genes were slightly underrepresented (*female biased*: *χ*^2^_1_ = 1.1, *P* = 0.30; *male biased*: *χ*^2^_1_ = 3.3, *P* = 0.07). In the somatic tissues, however, we found strong evidence that female-biased genes were enriched among the DE genes of E and L regimes, but not male-biased genes (FB: *χ*^2^_1_ = 263.4, *P* < 0.0001; MB: χ^2^_1_ = 2.9, *P* = 0.10). We also observed a strong skew in the somatic tissues in which sex showed evidence of expression divergence of sex-biased genes. Of the 143 female-biased DE genes, 85% were significant in females and only 22% in males. The same pattern was true for the 103 male-biased genes, with 87% being significant in females and only 38% in males.

We compared the median expression level between the E and L regimes in each sex, separately for all the female (FB)-, male (MB)-, and un-biased (UB) genes that showed evidence of expression divergence between the lines (fig. 5*c–f*). In the reproductive tissues, females did not differ in the median expression of FB genes (median test: *Z* = 1.24, *P* = 0.22, 95% confidence interval [CI] = −0.09, 0.38), but E females showed a significantly lower average expression of UB and MB genes (*Z* = −2.26, *P* = 0.024, 95% CI = −0.62, −0.08; *Z* = −3.86, *P* = 0.00011, 95% CI = −0.75, −0.32, respectively). In males, there were no average expression differences between the E and L in either type of sex-biased genes (FB genes: *Z* = −0.48, *P* = 0.64, 95% CI = −0.31, 0.18; MB genes: *Z* = −0.40, *P* = 0.68, 95% CI = −0.31, 0.21), but similar to females, the UB genes had a lower average expression in the E males compared with the L (*Z* = −2.67, *P* = 0.0075, 95% CI = −0.61, −0.10). In the somatic tissues the patterns were different. E females showed a significantly higher average expression of FB genes compared with the L, whereas the reverse was true in males (females: *Z* = 3.94, *P* < 0.0001, 95% CI = 0.30, 0.86; males: *Z* = −3.94, *P* = 0.0001, 95% CI = −0.70, −0.20). MB genes were expressed at a lower level in the E females relative to the L (*Z* = −2.62, *P* = 0.0088, 95% CI = −0.92, −0.093), but no significant difference was observed in males (*Z* = −1.57, *P* = 0.12, 95% CI = −0.88, 0.17). UB genes showed no average difference between the regimes in either sex (females: *Z* = −0.83, *P* = 0.40, 95% CI = −0.27, 0.087; males: *Z* = −1.81, *P* = 0.070, 95% CI = −0.28, 0.020). Although the degree of sex bias was highly correlated in the reproductive tissues of the abdomen between the E and L lines (*r* = 0.91, 95% CI = 0.91–0.92, *t* = 184.33, df = 6,677, *P* < 0.0001), it is less strong in the head and thorax (*r* = 0.58, 95% CI = 0.53–0.62, *t* = 21.72, df = 918, *P* < 0.0001), in line with a disproportionate divergence of female-biased genes in the somatic tissues.

Taken together, these patterns suggest a desexualization of the somatic transcriptome in the L life history regime relative to the E, driven predominantly by greater changes in female-biased genes in females. In the reproductive tissues also male-biased genes are less sex biased in the L, also largely due to changes in female expression.

### Functional Enrichment Analysis

GO term enrichment analyses revealed more significant terms among the genes differently expressed between E and L females than in males (supplementary Table S4 and fig. S2, Supplementary Material online). In female reproductive tissues, the enriched biological processes included for example ATP synthesis and mitochondrial electron transport, mitosis and defense response to bacteria and fungus, DNA integration, and snRNA import to the nucleus. In males, only a single term was significant—cyclic nucleotide biosynthetic process. In female somatic tissues, the significant terms included defense response, DNA integration, DNA-mediated transposition, and chitin-based cuticle development. In the male somatic tissues, the significant terms were related to mitosis and DNA integration.

### Lifespan Candidate Gene Differential Expression

We identified *A. obtectus* orthologs of proteins experimentally implicated to influence lifespan in *Drosophila melanogaster*. Three hundred and seventy-four significant hits were detected, corresponding to 157 *D. melanogaster* genes due to multiple isoforms. There were also multiple *A. obtectus* translated transcripts corresponding to each *D. melanogaster* protein, of which 122 were to unique predicted genes. The average bitscore for these blast hits was 554. Out of these 122 genes, 28 were DE between E and L with *q*-value <0.05, in a manner depending on the sex and tissue. This is a higher proportion than expected by chance when compared with the proportion of significantly DE genes out of those analyzed (*χ*^2^_1_ = 23.325, *P* < 0.0001). Ten and 8 genes were significant between E and L in the reproductive tissues, whereas 13 and 8 genes were significant in the somatic tissues of females and males, respectively (supplementary Table S3, Supplementary Material online).

## Discussion

We investigated how divergent EE of reproductive timing and longevity have affected transcriptome evolution in each sex. Overall, both sexes have diverged in lifespan, although females more so than males (fig. 1). In accordance, gene expression changes between the lines are more numerous in females (fig. 4). The greater female lifespan evolution reflects a stronger net selection on females. Although both sexes have adapted for either immediate or delayed reproduction, the EE regimes are less divergent in males, because due to female sperm storage capacity males can contribute to next generation even after death (Parsons and Credland 2003). In fact, it is difficult to envision an evolutionary scenario where divergent selection on lifespan is not sex specific at least to some extent, due to sexual dimorphism in reproductive roles and life histories. This suggests that evolutionary sex specificities similar to the ones documented here may be the rule rather than the exception.

The EE regimes have altered sex-biased expression patterns. Females and males of the long-lived L beetles are far more similar in gene expression, especially in the somatic tissues, than the short-lived E beetles. In addition to the life history regimes per se, the EE regimes have inadvertently altered the mating system, which is also predicted to affect sex-specific expression evolution. The temporal window for multiple mating is restricted in E lines (due to a female refractory period of 2 days in this species; Šešlija et al. 2009). In addition, L females have evolved a higher remating rate (Šešlija et al. 2009), likely to extract water and nutrients from male ejaculates to extend their lifespan (Arnqvist et al. 2022). Consequently, E males have evolved under relaxed sperm competition compared with the L males, whereas selection on females from mating interactions would also differ between the regimes. Based on the mating system and sexual selection, we would however expect more evolved differences in the reproductive than somatic tissues. We find instead that evolutionary history explains more variance in gene expression in the somatic tissues (fig. 3), and more genes are significantly DE in these than in the reproductive tissues (fig. 4). The monogamous mating system would also predict lower relative sex differences in the E lines due to more sexually concordant selection (Hollis et al. 2014), and not greater like we observed here. The patterns therefore point to a more direct role of the divergent life history regimes. Reduced sexual dimorphism in physiology (metabolic rate) is associated with adaptation to slower pace of life and longer lifespan across seed beetles (Arnqvist et al. 2022), echoing the gene expression divergence we observed in a micro-evolutionary scale.

The lower sex-biased expression in L lines is mostly due to changes in females that are masculinized relative to the E, due to expression divergence in both female- and male-biased genes (fig. 5). Because of our experimental design, we do not know the ancestral state of sex bias. The E line life history is closer to natural populations of *A. obtectus*, which could suggest that either the ancestral state is more intermediate or the reduced sex bias of the L is derived. The lowered feminization/increased masculinization could further imply that in order to delay reproductive senescence, the L females have concomitantly delayed some aspect of sexual maturation. L females are capable of reproducing at early ages (Tucić et al. 2004), but have a lower fecundity during the first 2 days after eclosion, while significantly greater late-life fecundity relative to the E (Tucić et al. 1998), in line with the predictions from life history theory. The lower feminization early in adulthood is thus likely a hallmark of female adaptation to late-life reproduction. Dissecting the mechanistic basis underlying the life history divergence and the changes in sex-biased transcriptome is an important avenue of future research.

Because lifespan is a complex trait, its evolution is subject to changes in trade-offs in multiple genetically correlated traits. In line with a large target of putative genes under divergent selection, we found that over 1,500 genes across tissues and sexes have significantly changed expression between the E and L beetles. It also means that with expression data alone it is not possible to tease apart genes directly associated with life history divergence from changes occurring due to linked selection. However, some insight can be gained from examining the functions of DE genes, and the overlap with known lifespan genes from different taxa. Most longevity genes have been identified using model organisms such as yeast, *C. elegans*, *Drosophila* and mice, by focusing on the analyses of large-effect mutants and transgenes, or examining gene expression patterns during aging. Unsurprisingly, segregating variation does not necessarily occur in genes with conserved effects on lifespan, as revealed by the limited overlap of genes affected by longevity selection in three populations of *D. melanogaster* (Fabian et al. 2018). Here, we did discover that *Drosophila* lifespan candidate genes are disproportionately common among the DE genes of E and L beetles. These include, for example, *FoxO*, *AMPK-γ*, heat-shock proteins *Hsp22* and *Hsp77*, and ecdysone receptor (*EcR*) (supplementary Table S3, Supplementary Material online). We provide an extended discussion on their role in lifespan divergence in supplementary Discussion, Supplementary Material online.

One common finding in *Drosophila* EE studies is that longer lifespan is associated with changes in immunity genes (Remolina et al. 2012; Carnes et al. 2015; Fabian et al. 2018). A connection between immunity and lifespan associated fitness traits has been hypothesized already for two decades (DeVeale et al. 2004), and in support, we also find an enrichment of genes related to “defense response to fungus” and “defense response to bacterium” (supplementary Table S4, Supplementary Material online) in the female reproductive tissues, whereas “defense response” was also the most significantly enriched group of genes in the female somatic tissues. This finding shows commonality across taxa and suggests that immunity investment may be a general mechanism underlying longevity, even though the overlap may be limited at the gene identity level (Fabian et al. 2018). The fact that E and L males do not notably differ in the expression of immunity genes may in part reflect their relatively lower divergence in longevity, but perhaps mostly the fact that trade-offs involving immunity show sex specificity in seed beetles (Bagchi et al. 2021).

It has also been long appreciated that genes with mitochondrial function are involved in aging across species (Cho et al. 2011; Tower 2015a; Sun et al. 2016; Kauppila et al. 2017; Srivastava 2017; Zhang et al. 2018; McGuire 2019; Son and Lee 2019), indicating a conservation of basic mechanisms (Tower 2015b). In accordance with the predictions from life history theory, growth, and reproduction seem to occur at the expense of the mitochondrial maintenance, leading to aging. Studies show, for example, downregulation of mitochondrial gene expression with age (McCarroll et al. 2004; Zahn et al. 2006; Landis et al. 2012). Uniparental inheritance of mitochondria also sets the stage for sex-specific selection on mitochondrial function (Gemmell et al. 2004; Tower 2006, 2015a). Some of the sex-specific functional abnormalities that occur with age include increased reactive oxygen species production and decreased ETC activity mediated by enzymes in the oxidative phosphorylation cascade (OXPHOS; Tower 2015a; Ventura-Clapier et al. 2017). Here, we find that genes involved in the ETC complex I activity (term “mitochondrial electron transport, NADH to ubiquinone”) are significantly enriched among the DE genes of E and L in the reproductive tissues, but again only in females (supplementary Table S4 and fig. S2, Supplementary Material online). This is also expected based on the previous findings of mitochondrial genetic effects in these lines. By creating mitonuclear introgression lines from the E and L beetles, Đorđević et al. (2017) showed that divergence in the epistatic interactions between nuclear and mitochondrial genes between the E and L affects especially ETC complex I activity in a sex-specific way. The ETC activity differences also explained a significant proportion of variation in aging-related life history phenotypes (Đorđević et al. 2017). In addition, there is also direct evidence that mitochondria have been under divergent selection favoring different mtDNA haplotype frequencies in the E and L lines (Stojković et al. 2017). At an organismal level, metabolic rate itself has indeed diverged between the E and L, and is 18% higher in the E beetles adapted to reproduction early in life (Arnqvist et al. 2017). Males generally show a higher metabolic rate and a faster decline in metabolic rate with age (Arnqvist et al. 2017). Together with the fact that we do not see significant differential expression of mitochondrial genes in males, these patterns are consistent with a genetic constraint due to the lack of mitochondrial adaptation in males. However, our results demonstrating gene expression divergence in females places mitochondrial function at the heart of life history trade-offs. The mitochondrial processes are multifaceted and deeply intertwined with many vital cellular processes. While essential as basic production sites for bioenergetics and macromolecules, they also regulate the communication and coordination of many vital physiological processes implicated in aging. Future work will be necessary to test the exact mechanism by which the expression changes we see early in adult life may affect lifespan divergence later in life.

## Conclusion

Here we have experimentally investigated the transcriptomic consequences of evolutionary divergence in the timing of reproductive investment and lifespan. By focusing on the early adult life, the expression differences we have detected reflect divergence in the timing of peak reproductive investment. Relative to the fast life history regime, we find that adaptation to late-life reproduction is associated with lower sexual dimorphism in gene expression due to females converging toward male expression patterns. Evolution of life histories is clearly a potent force driving expression evolution of sex-biased genes. In line with the life history theory, the functional categories of the DE genes implicate that reproductive timing is intimately linked with processes affecting longevity, including immunity and mitochondrial energy production.

## Materials and Methods

### Experimental Evolution

A base population of *A. obtectus* for the EE lines was established in 1983 by mass mating three local subpopulations collected near Belgrade (Tucić et al. 1996). The base population was maintained at a large size (*N* = 5,000) on *Phaseolus vulgaris* bean seeds in the laboratory for 3 years (27 generations) prior to the establishment of the EE lines. The EE lines were created simultaneously in 1986 by placing beetles under two divergent EE regimes. Under the E life history regime, the beetles were allowed to freely mate and lay eggs for the first 48 h of their adult life, after which the adults were discarded and the offspring left to develop inside the beans. Under the L regime, the beetles could freely mate but egg laying was permitted only after 10 days of adulthood, by withholding the oviposition substrates (bean seeds) until then. This represents a bi-directional EE design, as peak reproduction normally occurs in this species during 3–6 days of adulthood (Parsons and Credland 2003). Each of the EE regimes was replicated with four independent lines. Each generation, the E lines was started with ∼400 newly emerged adults (with ∼1:1 sex ratio) placed in rearing jars provided with a surplus of beans. Each L line was started with 1,000 adults kept together in 10 separate vials (∼100 individuals per vial) without beans to suppress egg laying. Beetles that survived to day 10 were then transferred to a common rearing jar provided with beans. Thus, only eggs laid after 10 days would contribute to the next generation. The EE lines were maintained in a dark incubator on *P. vulgaris* beans and under adult aphagy at 30 °C. More detailed descriptions of these lines can be found in Lazarević et al. (2012) and Tucić et al. (1996, 1997).

### Sample Preparation for RNA-Seq

The EE lines were kept under common garden conditions (i.e., no imposed selection on life histories due to unrestricted access to beans and mates for the entire life) for three generations prior to this experiment, after 193 and 282 generations of EE evolution for L and E beetles, respectively. This was primarily done to eliminate any transgenerational environmental effects that could differently influence the experimental beetles. We collected adult female and male beetles (virgin and socially naïve) 24 h after their emergence from beans (larval host). This time point corresponds to the peak of reproduction in the E regime, and therefore represents the age at which divergent selection has been strongest. After this age, the E beetles have no reproductive life under EE conditions. The beetles were first flash-frozen with liquid nitrogen and then the primary reproductive (abdomen) and non-reproductive (head and thorax, hereafter referred to as somatic) tissues were separated on ice. Six beetles per sex, tissue, and line were pooled and total RNA extracted from each pooled sample using RNeasy Mini Kit (Qiagen), following the manufacturer's protocol, according to the manufacturer's guidelines. The RNA quality and quantity were checked using Nanodrop, Bioanalyzer, and Qubit.

### Genome Sequencing, Assembly and Annotation

Briefly, for the genome assembly, we used a line of *A. obtectus* (that originates from the same wild population as the E and L), subjected to five consecutive generations of inbreeding (full-sib crosses) prior to sequencing. Only males of this inbred line were sequenced. Samples of whole-body genomic high-molecular-weight DNA were extracted (10 males per sample) and submitted to long-read sequencing using PacBio. Extractions were made using QIAGEN Genomic-tip 20/G, according to the manufacturer's protocol. Library preparation was done according to manufacturer's instructions. Sequencing was performed using 21 SMRT cells on a Sequel I system. The libraries and sequencing were done by the SNP&SEQ Technology Platform at Uppsala University.

Sequencing yielded a total 8,655,274 reads with an average read length of 10,176 bp (read length N50: 16,250 bp) which corresponds to an average genomic coverage of ∼80×. The genome was assembled using FALCON v 0.5.0 (https://github.com/PacificBiosciences/FALCON/) with default parameters. The genome completeness was assessed with BUSCO (v3.0.2b; Simão et al. 2015), using the insecta_odb9 gene data set. The genome annotation service at the National Bioinformatics Infrastructure Sweden (www.nbis.se) carried out the genome annotation, using a comprehensive MAKER3 pipeline (Holt and Yandell 2011). See the supplementary Methods, Supplementary Material online and methods for a detailed description of the assembly and annotation.

### Transcriptome Sequencing, Assembly and Annotation

We used the four replicate populations of the EE regimes as our biological replicates (i.e., E 1–4, L 1–4). Each sample type was sequenced as two technical replicates, which were subsequently merged before mapping, resulting in 32 libraries used for the analysis (8 lines × 2 tissue types × 2 sexes). The sequencing was performed using Illumina HiSeq 2500 sequencing V4 technology with a maximum read length of 2 × 125 bp. The paired-end libraries were first prepared using the TruSeq stranded mRNA Sample Preparation kit according to the manufacturer's guidelines (Illumina 2013). The library generation and sequencing were performed by the SNP&SEQ Technology Platform at Uppsala University. In total, 743 million pairs of reads were generated. 705 million clean pairs (94.8%) were retained after quality filtering (described in the supplementary material and methods) and used for the assembly. We used a genome-guided assembly of the transcriptome, by mapping the reads from the 32 samples to our *A. obtectus* reference genome described above. See the supplementary material and methods for a detailed description of the assembly and annotation of the transcriptome.

### Analysis of Gene Expression Differences

Analyses of gene expression were performed using the edgeR (v.3.32.0; Robinson et al. 2009) package within Bioconductor (Gentleman et al. 2004), using R v. 4.0.3 (R Core Team 2019). The count data were filtered to include only genes where minimum of three samples showed expression of over 2 counts per million reads (cpm). Following the pipeline of edgeR, we first normalized the data by computing scaling factors using the trimmed mean of *M* values. We then used a generalized linear model with likelihood ratio tests, where a negative binomial model is fitted with a Cox-Reid profile adjusted likelihood ratio method to estimate tag-wise dispersion. In all analyses, we used a statistical significance threshold of 5% false discovery rate (Benjamini and Hochberg 1995). In addition, to call a gene DE between the sexes and the EE regimes, we required a minimum of 2-fold difference in expression (i.e., absolute log_2_FC > 1), to safeguard against differences due to possible tissue scaling effects (Montgomery and Mank 2016). We tested for overrepresentation of Gene Ontology terms (Biological Process and Molecular Function) among DE genes with the GOstats package (v. 2.56.0), using a conditional hypergeometric test with a *P*-value cutoff > 0.05 (Falcon and Gentleman 2007). The *P*-values were adjusted for multiple testing with false discovery rate. We defined the gene universe as all expressed genes in a given condition.

In our analytical pipeline, we first characterized the degree of similarity in expression of all genes between the replicate populations experiencing the same EE regime, by calculating multivariate repeatabilities of the evolution of gene expression using a geometric approach (Nakagawa and Schielzeth 2010). From a principle component analysis of trimmed mean of *M* values normalized expression data based on the covariance matrix across all genes, we retained the first 16 principle components. Following the ordination of all 32 samples as well as all 8 group specific centroids (EE regime × sex × tissue type) in this 16-dimensional space, we calculated repeatability as the Euclidean distance (ED) between the E and L centroids divided by the sum of the ED between the E and L centroids and the average ED between the samples and their group specific centroid. This intuitive measure thus expresses the disparity of the four replicate lines relative to the difference between E and L lines. For example, a repeatability of *R* = 0.66 of gene expression in the head/thorax of males in the E regime means that replicate E lines are half as different from their own average as are E lines from L lines.

Second, we tested for differential expression between the E and L regimes, contrasting separately the sexes and tissues, as well as testing sex-by-selection interactions. Third, we examined sex-biased expression within each EE regime, separately for each tissue. Proportion tests were used for assessing overrepresentation of sex-biased genes among the genes that had significantly diverged between E and L females and males, to test how each sex contributes to the evolution of sexually dimorphic expression. For this, a gene was defined as sex biased when significantly DE between the sexes in either EE regime. For the female-, male-, and un-biased genes that were significantly differently expressed between the E and L regimes (in either sex), we also tested whether they collectively showed any median expression difference, with the aim to test how each sex contributes to putative concomitant changes in sexualization of the transcriptome. Lastly, we compared the correlation of sex bias between the E and L regimes, separately in each tissue.

In addition, we tested whether any known lifespan candidate genes from *D. melanogaster* were DE between the E and L lines, using the Ageing Gene Database (Tacutu et al. 2018). The translated aging gene sequences were downloaded from FlyBase (https://flybase.org) and the translated *A. obtectus* transcripts were blasted against these using NCBI protein blast. We searched for orthologs of 389 *D. melanogaster* proteins using the default settings (*e*-value threshold of 0.001). The best hit for each protein was chosen by selecting on *e*-value and bit score (Altschul et al. 1990).

## Supplementary Material

evac177_Supplementary_Data

## Data Availability

The annotated genome assembly, along with sequence data, is available from the European Nucleotide Archive (ENA) under the project ID: PRJEB51445 (genome accession ID: GCA_933228535). Raw RNA-Seq data are deposited in FASTQ format to the NCBI Sequence Read Archive database (SRA) under the BioProject accession number PRJNA492259. The assembly have been deposited at GenBank under the accession number GGYI00000000.1.
