# Supplementary material for: Experimental Life History Evolution Results in Sex-specific Evolution of Gene Expression in Seed Beetles
Source: Genome Biol Evol. 2022 Dec 21;15(1):evac177. doi: 10.1093/gbe/evac177 (PMC9830990; doi:10.1093/gbe/evac177)
Supplement: evac177_Supplementary_Data [file evac177_supplementary_data.zip › Immonenetal_SupplementaryInformation.pdf]

Experimental life history evolution results in sex-specific evolution of gene expression in seed beetles

Elina Immonen, Ahmed Sayadi, Biljana Stojković, Uroš Savković, Mirko Đorđević, Johanna Liljestrand-Rönn, R. Axel Wiberg, Göran Arnqvist

Supplementary material and methods

Supplementary methods
1. *Acanthoscelides obtectus* genome sequencing and assembly
2. Genome annotation
3. Transcriptome sequencing and assembly
4. Transcriptome annotation

Supplementary figures
- Figure S1
- Figure S2

Extended Discussion

Genome sequencing and assembly

For genome sequencing and assembly, we first subjected a line of *A. obtectus* (originally from the same population as the E and L lines) to five consecutive generations of inbreeding by propagating a single female mated to a full sib brother. Males of this inbred line were subsequently used for sequencing.

Samples of whole-body genomic high-molecular-weight DNA was extracted (10 males per sample) and submitted to long-read sequencing using PacBio. Extractions were made using QIAGEN Genomic-tip 20/G, according to the manufacturer's protocol. High molecular weight DNA was sheared using the Megaruptor 2 system (Diagenode) with a 25 kb target. Size-selection with a 15 kb cut-off was done using the Blue Pippin system (SAGE). SMRTbell Template Prep Kit 1.0 was used for library construction according to manufacturer's instructions. Sequencing was performed using 21 SMRT cells on a Sequel I system, with 20 hr movies and V2 chemistry.

Our sequencing effort yielded in total 8,655,274 reads with an average read-length of 10,176 bp (read-length N50: 16,250 bp) which corresponds to an average genomic coverage of approximately 80X. The genome was then assembled using FALCON v 0.5.0 (https://github.com/PacificBiosciences/FALCON/) with default parameters, based on the PacBio read data. The assembly was subsequently error-corrected by one round of Arrow (SMART portal) based on re-alignment of the full set of PacBio reads.

The resulting polished genome assembly is 1.1 Gb in total size, contains 6,654 contigs with an N50 of 791 kb. Genome completeness was assessed with BUSCO (v3.0.2b) (Simão et al. 2015), using the the insecta_odb9 gene dataset. This showed a very high fraction of well-assembled genes in the assembly (Table S1).

**Table S1: Statistics for the *A. obtectus* genome assembly.**		
	**Number**	**%**
Nucletides (size)	1,103,332,300	
Sequences	6,654	
GC content (%)	37.5	
N50	791,033	
Total BUSCO groups searched	1,658	
Complete BUSCOs	1,624	98
Complete single copy BUSCOs	1,447	87.3
Complete duplicated BUSCOs	177	10.7
Fragmented BUSCOs	13	0.8
Missing BUSCOs	21	1.2

Genome annotation

The genome annotation service at the National Bioinformatics Infrastructure Sweden (www.nbis.se) carried out the genome annotation, using a comprehensive MAKER3 pipeline (Holt and Yandell 2011) as detailed below. We created a species specific repeat library modeled using the RepeatModeler package (1.0.8) (Smit and Hubley R. 2010). All candidate repeats modeled by RepeatModeler were vetted against our protein set (minus transposons) to exclude any nucleotide motif stemming from low-complexity coding sequences. From the repeat library, identification of repeat sequences present in the genome was then performed using RepeatMasker (4.0.3) (Smit et al. 2010) and RepeatRunner (Yandell 2006). In total 1,235,984 repeats were masked, constituting a total size of 691,912 kb (62.7 % of the total assembly).

A first round of annotation was performed with MAKER3 using both (1) curated protein sequences collected from the Uniprot Swiss-Prot database (Magrane and Consortium 2011) and (2) the extensive amount of transcriptome data generated in the current study, in the form of both a single *de novo* assembly generated with Trinity (Grabherr et al. 2011) and the 32 guided assemblies made with StringTie (Pertea et al. 2015). This evidence-based gene build resulted in a first “release candidate” gene set (rc1) with 20,682 gene models.

The evidence-based annotation is limited by the available sequence data, which can lead to fragmented gene models and missed genes. To prevent this from happening, we next performed an *ab initio* evidence-driven gene build, where available protein and transcript evidence is used to help and guide *ab initio* tools during their prediction processes. From the first evidence-based gene build (rc1), we selected a high-confidence set of genes used to train the *ab initio* tools Augustus 2.7 (Stanke et al. 2006) and Snap 2006-07-28 (Korf 2004). We also trained GeneMark-ET 4.3 (Lomsadze et al. 2014), which is a self-trained method integrating RNA-seq evidence using the *junctions.bed* file from Tophat. The *ab initio* evidence-driven annotation was performed with MAKER3, using both the output HMM-models from the trained *ab initio* tools (Augustus, Snap, and Genemark-ET) and the same evidence data as used previously. We also used EVidenceModeler (EVM) (Haas et al. 2008), which allowed us to perform gene models based on the best possible set of exons produced by the other *ab initio* tools, and choose the most consistent according to the available evidence. The *ab initio* evidence-driven gene build (rc2) contained 35,123 gene models.

Finally, all *ab initio* gene models (rc2) that mapped within an empty locus in the evidence-driven annotation (rc1), was added to rc1 to create our final build (rc3), containing 38,104 gene models. The details of the annotation are given in Table S2.

Table S2: Annotation statistics (rc3) for the *A. obtectus* genome.	
Number of protein-coding genes	38,104
Number of mRNA	68,812
Average number of exons per mRNA	5.6
Average exon length	347
Average intron length	3,141
Average CDS length	1,113
Fraction of the genome covered by genes (%)	44.2

For the final gene build (rc3), we inferred putative functions for all genes. To this end, we first predicted functional domains using InterProscan 5.21-60 (Jones et al. 2014) to retrieve functional information from Interpro (21 different sources). Functional annotations were thus assigned to 26,344 of the predicted coding genes and to 46,333 of the predicted mRNAs. Each predicted protein sequence was also blasted against the Uniprot/Swissprot reference data set in order to infer, when available, the gene and protein name. The inference was made using the best blast hit approach, i.e. using the best hit with a maximum e-value cut-off to 1e-6. This made it possible to associate gene names to 15,387 protein sequences. In addition, 721 tRNA genes were annotated and added to rc3 through *tRNAscan* 1.3.1 (Lowe and Eddy 1997).

The annotated genome assembly, along with sequence data, is available from the European Nucleotide Archive (ENA) under the project ID: PRJEB51445 (genome accession ID: GCA_933228535).

Transcriptome sequencing and assembly

We sequenced 64 samples in total. Each sample type was sequenced as two technical replicates which were subsequently merged before mapping, resulting in 32 libraries used for the analysis. The sequencing was performed using Illumina HiSeq 2500 sequencing V4 technology with a maximum read length of 2x125 bp. The paired-end libraries were prepared using the TruSeq stranded mRNA Sample Preparation kit according to the manufacturer's guidelines (Illumina 2013). The library generation and sequencing were performed by the SNP&SEQ Technology Platform at Uppsala University. In total, 743 million pairs of reads were generated.

The raw reads were checked for quality using the FastQC (v.0.11.5) tool (Andrews 2010) and low quality bases/reads were removed using Cutadapt (v.1.9.1) (Martin 2011) and Trimmomatic (Bolger et al. 2014) (v.0.36). Illumina adapters were removed from raw reads using Cutadapt. Low quality bases, with average phred quality score lower than 20 were dropped. Reads were scanned with a 4 base wide sliding window, and leading or trailing bases at the 3' and at the 5' ends of the reads were trimmed using Trimmomatic. Reads shorter than 50bp were also discarded. On average all reads obtained a phred score

> 37, and a total of 705 million clean pairs of reads (94.8%) were used for the transcriptome assembly.

The transcriptome was assembled by mapping the reads from the 32 biological samples to our *A. obtectus* reference genome described above, using HISAT (Pertea et al. 2016). The mapping rate of the reads from each sample to the genome was 80-92%. Mapped reads were then assembled to transcripts using StringTie tool (Pertea et al. 2015). Each sample was first assembled separately and subsequently merged to form one uniform transcriptome assembly using StringTie merge function. Gene and transcript abundancies were also estimated with StringTie. Read count data was extracted from StringTie output using an in-house python script (prepDE.py).

Transcriptome annotation

All transcripts were first blasted against the NCBI (non-redundant) protein database (Nr) (ftp://ftp.ncbi.nih.gov/blast/db/ 06-06-2018) and the Swissprot-Uniprot database, with an E-value cut-off of 10–5 (Altschul et al. 1990). Top blast hits were then fed to the Blast2go software (version 5.1) (Conesa et al. 2005) and to the Trinotate tool (https://trinotate.github.io/), for further annotation. Blast2GO and Trinotate help to extract all the information from other databases to attribute a function or a domain to our transcripts, using a homology search against several different databases, such as the Kyoto Encyclopedia of Genes and Genomes (KEGG) (Kanehisa et al. 2012), GO database (Gene Ontology) (Ashburner et al. 2000), PFAM (Finn et al. 2016) and EggNog (Powell et al. 2012). Also several other prediction tools were incorporated into the pipeline, including InterProScan (Zdobnov and Apweiler 2001) (Zdobnov and Apweiler 2001)for Blast2GO, and SignalP (v.4.1) that predict signal peptide cleavage sites (Petersen et al. 2011), Tmhmm (v.2.0c) that predicts transmembrane helices in proteins (Krogh et al. 2001), as well as Rnammer (v.1.2), which predicts ribosomal RNA (Lagesen et al. 2007).

The gene completeness of the assembled transcriptome was assessed using the BUSCO (Benchmarking Universal Single-Copy Orthologs) library (http://busco.ezlab.org/) (Simão et al. 2015), by scanning the transcriptome for the presence of known highly conserved orthologous genes (978 single-copy metazoan orthologs). Gene open reading frames (ORFs) were predicted using Transdecoder v.3.1.1 (http://transdecoder.sourceforge.net/). ORFs longer than 100 amino acids were retained.

The assembled transcriptome contains 50,481 transcripts, corresponding to 32,006 genes. The GC content is 39.63%, which is consistent with other beetle genomes (i.e. *D. ponderosae* at 36% (Keeling et al. 2013), *T. castaneum* at 33% (Tribolium Genome Sequencing Consortium 2008). The assembly has an N50 length of 4117 bases and a mean transcript size of 2089.6 bases. Transcript length ranges from 200 bases to 50358 bases, with 26681 transcripts being >1 kb and 1026 being >10 kb. 77% of the genes are represented as single isoforms. In addition to the primary statistics, we assessed the quality of the transcriptome assembly using the BUSCO tool, and detected 945 (97%)

complete BUSCO-list genes in our transcriptome, of which 338 (35%) were duplicate hits. The number of duplicates may reflect allelic variation (heterozygosity) between our samples, gene duplications and/or alternative splicing events. There were very few fragmented or missing genes (20 and 13, respectively).

In our annotation pipeline, we detected 22,794 (71%) genes in the protein (Nr) database. 96% of the blast hits belonged to Metazoan taxa, while the rest had a very low sequence similarity with viruses, bacteria, fungi and viridiplantae. Most of the best blast hits belonged to three beetle species (41%, *Anoplophora glabripennis*; 23%, *Lepinotarsa decemlineata*; 6%, *Tribolium castaneum*) belonging to the same infraorder (Cucujiformia) as *A. obtectus*. The remaining blast hits showed sequence similarities to other insects in a majority of the cases, and, reassuringly, most of these (73%) have a greater than 65% sequence identity with the best Nr-protein database hit. Finally, we tested the ability of each gene to reconstruct complete open reading frames (ORFs). We obtained 17,553 genes (11,275 with complete ORFs) with an average ORF length of 505 amino acids. No less than 96% of the genes with ORFs show a blast hit in the Nr database, with an average length of 516 amino acids. All these statistics validate the quality of our assembly.

The COG classification (Clusters of Orthologous Groups) shows that a total of 3,570 genes were assigned to 25 COG functional categories. The largest group is represented by the cluster for general function prediction (21%), followed by signal transduction mechanisms (9%), translation, ribosomal structure and biogenesis, posttranslational modification, protein turnover, chaperones, amino acid transport and metabolism, lipid transport and metabolism carbohydrate transport (7%).

Raw RNA-Seq data is deposited in FASTQ format to the NCBI Sequence Read Archive database (SRA) under the BioProject accession number PRJNA492259. The assembly have been deposited at GenBank under the accession number GGYI00000000.1.

Supplementary Figures

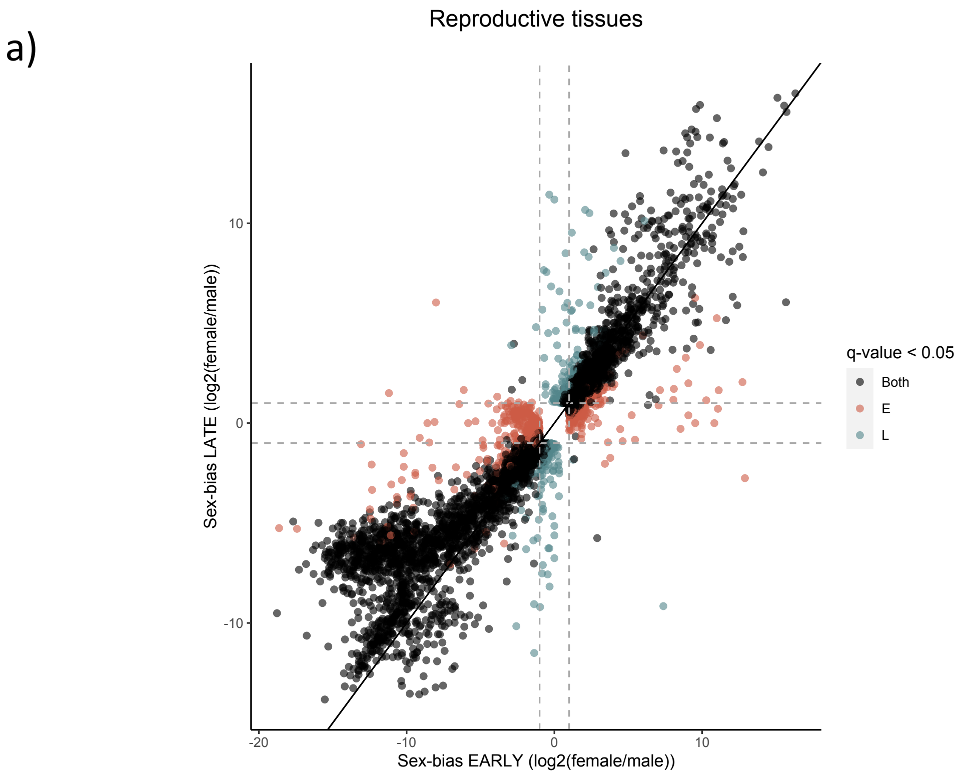

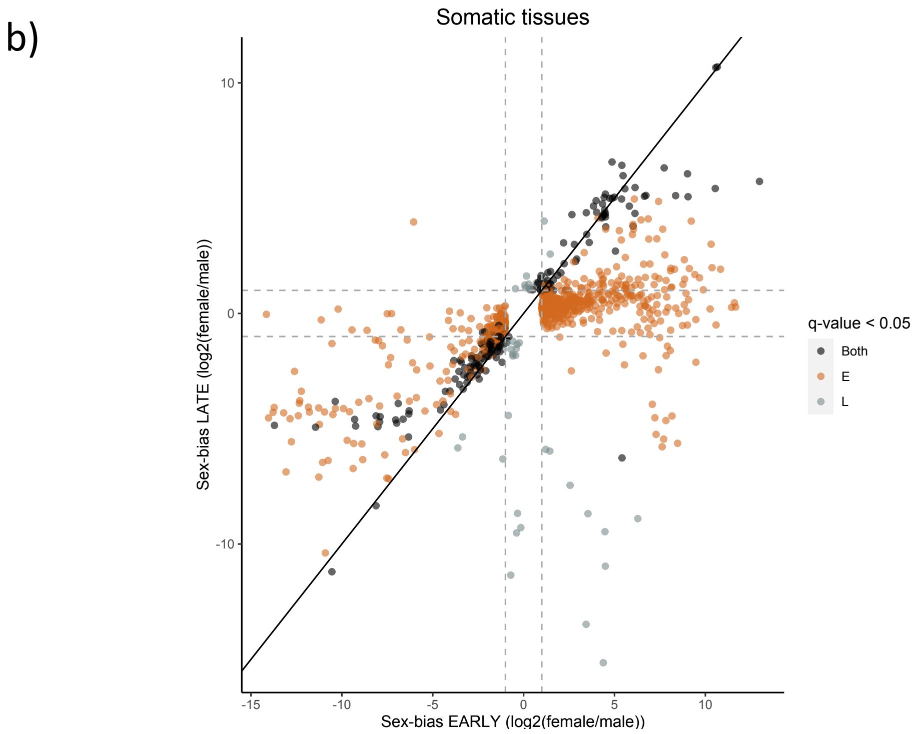

Figure S1. Correlation of sex-biased expression in the (**E**)arly and (**L**)ate life history selection lines in the **a)** reproductive and **b)** somatic tissues. Genes with significant sex-bias in both selection regimes are shown in black, and those significant only in the E or L regime in orange and grey, respectively.

a)

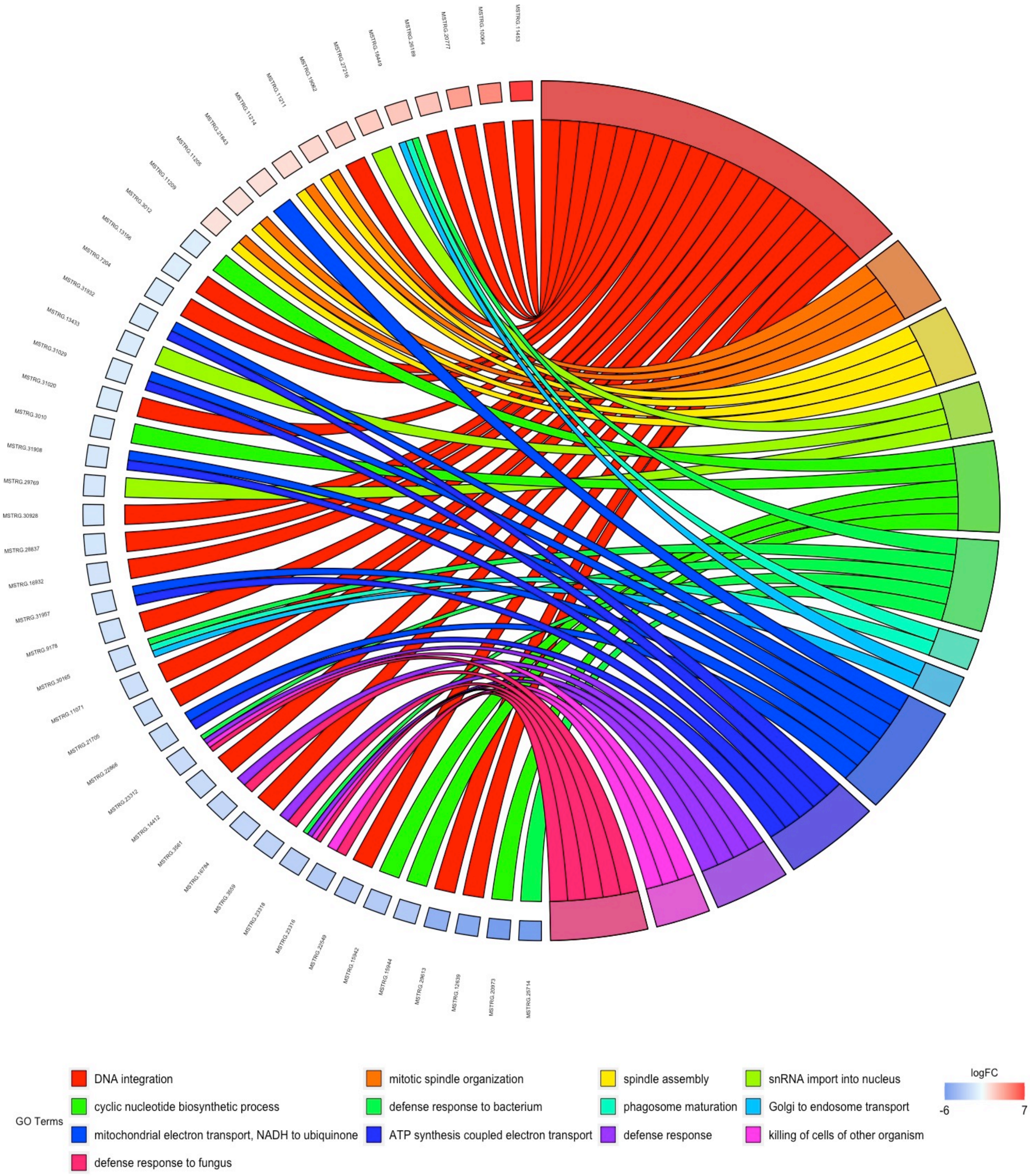
DNA integration
mitotic spindle organization
spindle assembly
snRNA import into nucleus
cyclic nucleotide biosynthetic process
defense response to bacterium
phagosome maturation
Golgi to endosome transport
GO Terms
mitochondrial electron transport, NADH to ubiquinone
ATP synthesis coupled electron transport
defense response
killing of cells of other organism
defense response to fungus
logFC
-6
7

b)

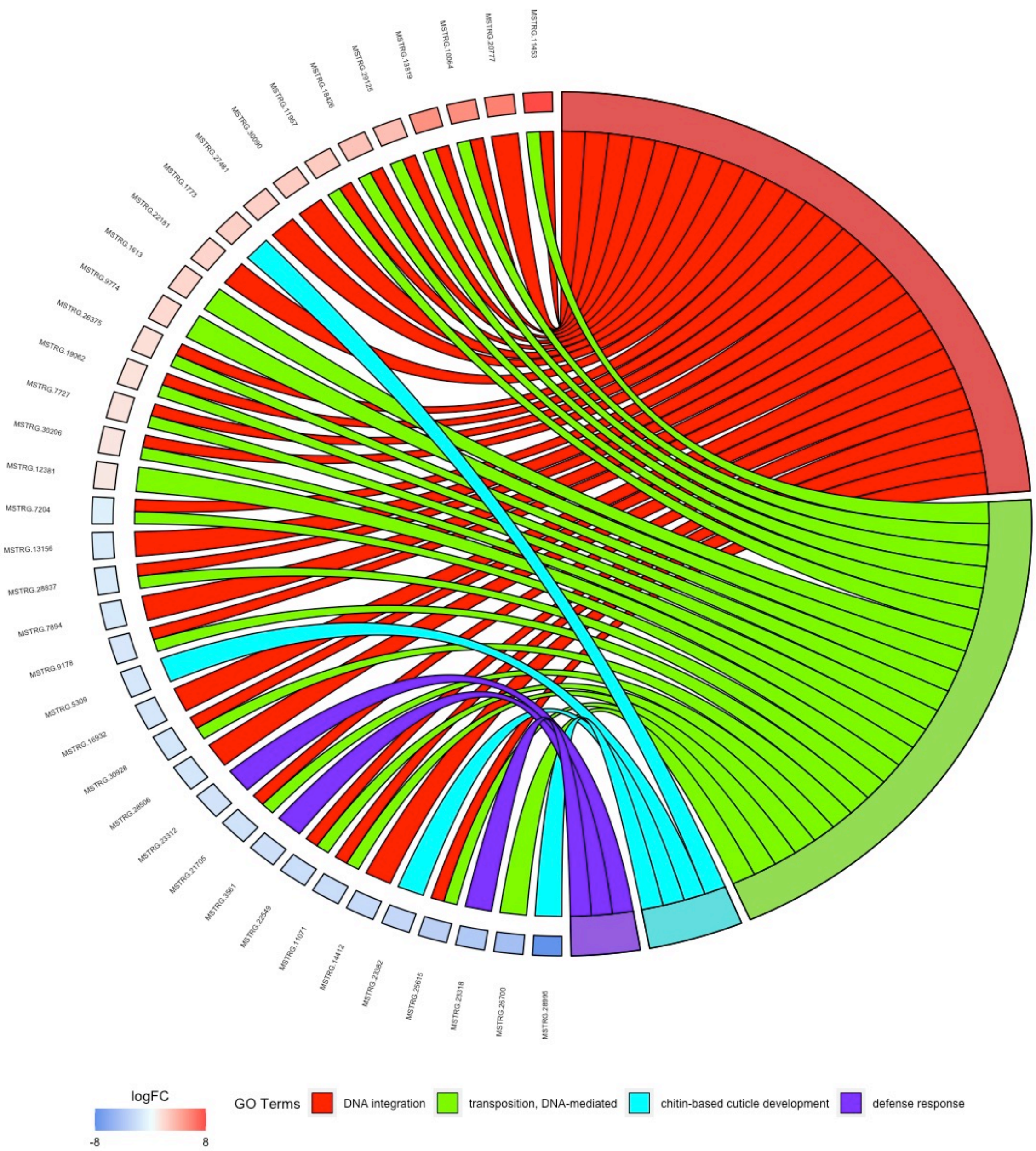
MSTRG.11453
MSTRG.20777
MSTRG.10064
MSTRG.13819
MSTRG.29125
MSTRG.18426
MSTRG.11957
MSTRG.30090
MSTRG.27481
MSTRG.1773
MSTRG.22181
MSTRG.1613
MSTRG.9774
MSTRG.26375
MSTRG.19062
MSTRG.7727
MSTRG.30206
MSTRG.12381
MSTRG.7204
MSTRG.13156
MSTRG.28837
MSTRG.7894
MSTRG.9178
MSTRG.5309
MSTRG.16932
MSTRG.30928
MSTRG.28506
MSTRG.23312
MSTRG.21705
MSTRG.3561
MSTRG.22549
MSTRG.11071
MSTRG.14412
MSTRG.23382
MSTRG.25615
MSTRG.23318
MSTRG.26700
MSTRG.28995
logFC
-8
8
GO Terms
DNA integration
transposition, DNA-mediated
chitin-based cuticle development
defense response

c)

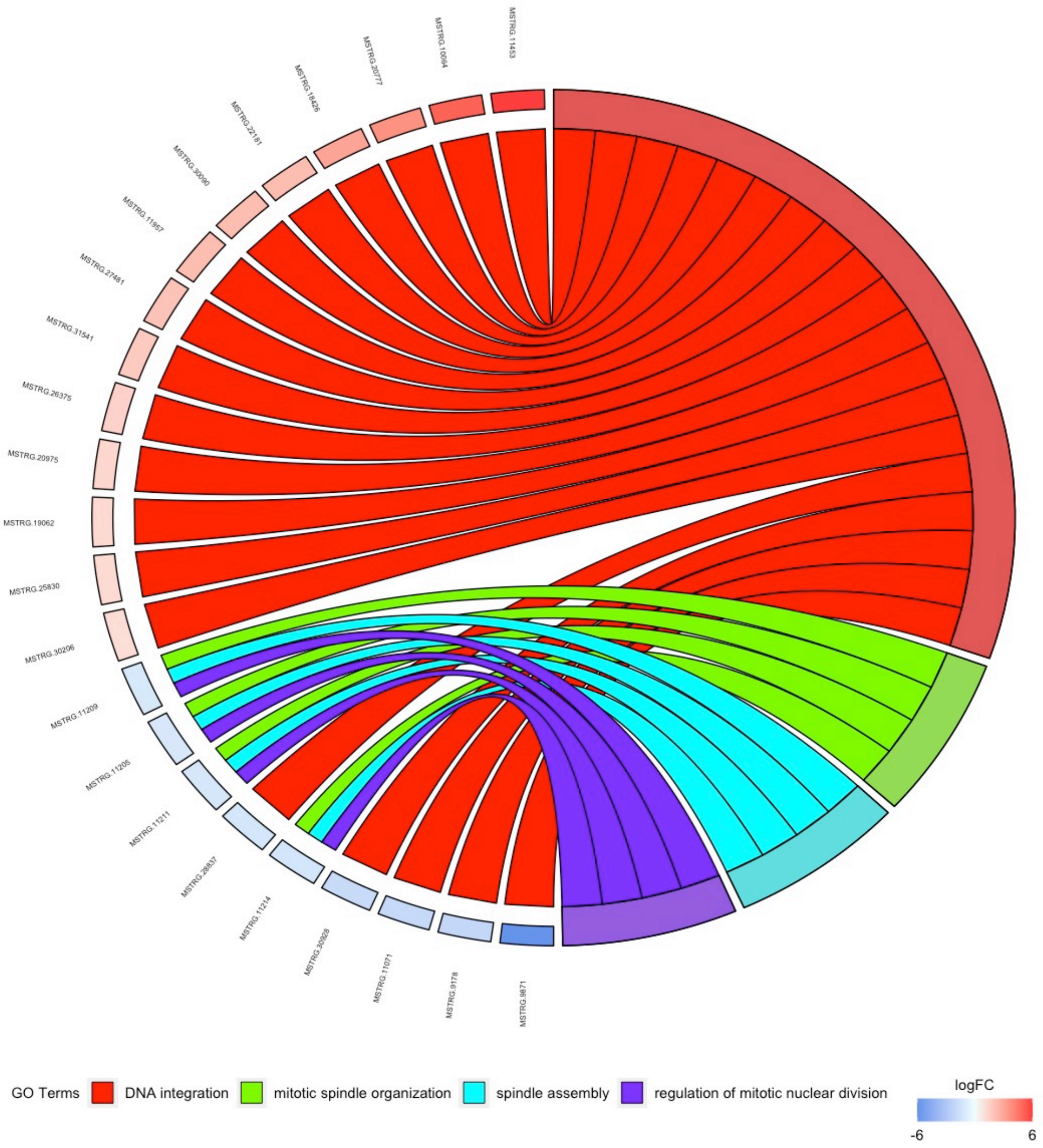

Figure S2. Chord diagram presenting the significantly enriched gene ontology terms (biological processes) among the differentially expressed genes between the E and L beetles, in the **a)** female reproductive tissues, as well as **b)** female and **c)** male somatic tissues. The ribbons represent genes under each GO category, and the outer circle the expression difference (positive logFC indicates a higher relative expression in the E beetles).

Extended Discussion

Most longevity genes have been identified using model organisms such as yeast, *C. elegans*, *Drosophila* and mice, by focusing on the analyses of large-effect mutants and transgenes, or examining gene expression patterns during ageing. We investigated whether *A. obtectus* orthologs of any known longevity genes in *Drosophila* may have diverged in expression due to experimental life history evolution, and found several interesting candidates.

The major evolutionarily conserved pathways that regulate lifespan include the insulin/insulin-like growth factor 1 signaling (IIS). It has been suggested that IIS may be under strong purifying selection and therefore less likely to contribute to segregating variation and thus contemporary longevity evolution (Fabian et al., 2018; Flatt & Partridge, 2018; Remolina et al., 2012). We did detect differential expression in the gene coding forkhead box protein O (FoxO), a transcription factor targeted by IIS. Overall, FoxO is involved in regulating several crucial cellular functions, including apoptosis, cell cycle, stress resistance, glucose and lipid metabolism and inflammation (Calnan & Brunet, 2008; Martins et al., 2016; Nakae et al., 2008; Peng, 2008). Inhibiting IIS kinases extends lifespan by transcriptional activation of FoxO and its target genes. Reduced FoxO pathway activity in turn impairs the maintenance of resistance against cellular stressors reducing longevity (Calnan and Brunet 2008; Greer and Brunet 2008; Martins et al. 2016). In line with this, higher FoxO gene expression was detected in the L female somatic tissues.

Another key ageing pathway involves highly conserved AMP-activated kinases (AMPK) composed of catalytic α subunit and regulatory β and γ subunits (Salminen and Kaarniranta 2012). AMPK regulate energy metabolism, coordinate several housekeeping mechanisms including autophagocytosis of damaged structures and alleviate stress by increasing tissue resistance (Salminen and Kaarniranta 2012; Salminen et al. 2016). The gene coding for SNF4Aγ, the *Drosophila* homolog of the AMPK γ subunit, showed a two-fold higher expression in the abdomen of E beetles in both sexes relative to the L, and over 1.6 times higher expression in the E male head and thorax tissues. AMPK pathway has been implicated in ageing in yeast (e.g. Friis et al., 2014), *C. elegans* (e.g. Mair et al., 2011) and *Drosophila* (e.g. Ulgherait et al., 2014) through regulating functions of several other anti-ageing pathways (Salminen and Kaarniranta 2012), including FoxO signaling (Greer et al. 2007b,a). Overall, AMPK shows a reduced expression with age in many organisms leading to lowered capacity to respond to stresses, which is thought to augment the aging process (Salminen et al. 2016). The fact that we find a reduced expression of the γ subunit gene in long-lived beetles at a young age could indicate energy conservation early in life. AMPK is activated when cellular energy levels are low, which generates insulin-sensitizing effects resulting in increased glucose uptake and enhanced fatty acid oxidation (Ruderman et al. 2013). Given that the L beetles have evolved longer lifespan under aphagous conditions that essentially requires starvation resistance, the lower AMPK expression could indicate some kind of reprogramming of metabolic homeostasis from the early age that preserves energy resources. Differences between E and L lines in the

temporal pattern of metabolic substrate use (Arnqvist et al. 2017) is consistent with this interpretation.

Another interesting class of ageing genes detected are heat-shock proteins (HSP) that protect cells from protein aggregation, misfolded proteins, cytotoxicity and apoptosis (Murshid et al. 2013). HSPs respond to stresses such as heat and oxidative stress, and ageing cells loose capacity to synthesize HSPs, essentially leading to a decline in protein quality control (Murshid et al. 2013). In *Drosophila*, aging has been associated with proteins Hsp22 and Hsp77 (Yang and Tower 2009). Here we find that genes coding each of these are expressed at over two-fold higher level in the long-lived L males in the somatic tissues, suggesting divergence in maintaining protein homeostasis and stress tolerance, possibly in the brain (Gestwicki and Garza 2012). Another interesting candidate gene only in male somatic tissues is the *Drosophila* ortholog for ecdysone receptor (EcR), which shows higher expression in the E beetles. EcR in *Drosophila* affects adult lifespan in sex-specific way (Tricoire et al., 2009). Ectysteroids are steroid hormones required in developmental transitions and metamorphosis, but in adult males they are involved in courtship, spermatogenesis and longevity regulation (Tricoire et al. 2009). A modest inactivation of EcR in *Drosophila* extends lifespan but only in males, echoing the pattern we detected (Tricoire et al. 2009).

References:

Altschul, S. F., W. Gish, W. Miller, E. W. Myers, and D. J. Lipman. 1990. Basic local alignment search tool. J Mol Biol 215.

Andrews, S. 2010. FastQC - A quality control tool for high throughput sequence data. http://www.bioinformatics.babraham.ac.uk/projects/fastqc/. Babraham Bioinformatics.

Ashburner, M., C. A. Ball, J. A. Blake, D. Botstein, H. Butler, J. M. Cherry, A. P. Davis, K. Dolinski, S. S. Dwight, J. T. Eppig, M. A. Harris, D. P. Hill, L. Issel-Tarver, A. Kasarskis, S. Lewis, J. C. Matese, J. E. Richardson, M. Ringwald, G. M. Rubin, and G. Sherlock. 2000. Gene ontology: Tool for the unification of biology.

Bolger, A. M., M. Lohse, and B. Usadel. 2014. Trimmomatic: A flexible trimmer for Illumina sequence data. Bioinformatics 30.

Calnan, D. R., and A. Brunet. 2008. The FoxO code.

Conesa, A., S. Götz, J. M. García-Gómez, J. Terol, M. Talón, and M. Robles. 2005. Blast2GO: A universal tool for annotation, visualization and analysis in functional genomics research. Bioinformatics 21.

Fabian, D. K., K. Garschall, P. Klepsatel, G. Santos-Matos, É. Sucena, M. Kapun, B. Lemaitre, C. Schlötterer, R. Arking, and T. Flatt. 2018. Evolution of longevity improves immunity in Drosophila.

Finn, R. D., P. Coggill, R. Y. Eberhardt, S. R. Eddy, J. Mistry, A. L. Mitchell, S. C. Potter, M. Punta, M. Qureshi, A. Sangrador-Vegas, G. A. Salazar, J. Tate, and A. Bateman.

2016. The Pfam protein families database: Towards a more sustainable future. Nucleic Acids Res 44.

Flatt, T., and L. Partridge. 2018. Horizons in the evolution of aging.

Friis, R. M. N., J. P. Glaves, T. Huan, L. Li, B. D. Sykes, and M. C. Schultz. 2014. Rewiring AMPK and Mitochondrial Retrograde Signaling for Metabolic Control of Aging and Histone Acetylation in Respiratory-Defective Cells. Cell Rep 7.

Gestwicki, J. E., and D. Garza. 2012. Protein quality control in neurodegenerative disease. P. *in* Progress in Molecular Biology and Translational Science.

Grabherr, M. G., B. J. Haas, M. Yassour, J. Z. Levin, D. A. Thompson, I. Amit, X. Adiconis, L. Fan, R. Raychowdhury, Q. Zeng, Z. Chen, E. Mauceli, N. Hacohen, A. Gnirke, N. Rhind, F. di Palma, B. W. Birren, C. Nusbaum, K. Lindblad-Toh, N. Friedman, and A. Regev. 2011. Full-length transcriptome assembly from RNA-Seq data without a reference genome. Nat Biotechnol 29:644–652.

Greer, E. L., and A. Brunet. 2008. FOXO transcription factors in ageing and cancer. P. *in* Acta Physiologica.

Greer, E. L., D. Dowlatshahi, M. R. Banko, J. Villen, K. Hoang, D. Blanchard, S. P. Gygi, and A. Brunet. 2007a. An AMPK-FOXO Pathway Mediates Longevity Induced by a Novel Method of Dietary Restriction in C. elegans. Current Biology 17.

Greer, E. L., P. R. Oskoui, M. R. Banko, J. M. Maniar, M. P. Gygi, S. P. Gygi, and A. Brunet. 2007b. The energy sensor AMP-activated protein kinase directly regulates the mammalian FOXO3 transcription factor. Journal of Biological Chemistry 282.

Haas, B. J., S. L. Salzberg, W. Zhu, M. Pertea, J. E. Allen, J. Orvis, O. White, C. R. Buell, and J. R. Wortman. 2008. Automated eukaryotic gene structure annotation using EVidenceModeler and the Program to Assemble Spliced Alignments. Genome Biol 9:R7.

Holt, C., and M. Yandell. 2011. MAKER2: an annotation pipeline and genome-database management tool for second-generation genome projects. BMC Bioinformatics 12:491.

Jones, P., D. Binns, H.-Y. Chang, M. Fraser, W. Li, C. McAnulla, H. McWilliam, J. Maslen, A. Mitchell, G. Nuka, S. Pesseat, A. F. Quinn, A. Sangrador-Vegas, M. Scheremetjew, S.-Y. Yong, R. Lopez, and S. Hunter. 2014. InterProScan 5: genome-scale protein function classification. Bioinformatics 30:1236–1240.

Kanehisa, M., S. Goto, Y. Sato, M. Furumichi, and M. Tanabe. 2012. KEGG for integration and interpretation of large-scale molecular data sets. Nucleic Acids Res 40.

Korf, I. 2004. Gene finding in novel genomes. BMC Bioinformatics 5:59.

Krogh, A., B. Larsson, G. von Heijne, and E. L. L. Sonnhammer. 2001. Predicting transmembrane protein topology with a hidden Markov model: Application to complete genomes. J Mol Biol 305.

Lagesen, K., P. Hallin, E. A. Rødland, H. H. Stærfeldt, T. Rognes, and D. W. Ussery. 2007. RNAmmer: Consistent and rapid annotation of ribosomal RNA genes. Nucleic Acids Res 35.

Lomsadze, A., P. D. Burns, and M. Borodovsky. 2014. Integration of mapped RNA-Seq reads into automatic training of eukaryotic gene finding algorithm. Nucleic Acids Res 42:e119–e119.

Lowe, T. M., and S. R. Eddy. 1997. tRNAscan-SE: A Program for Improved Detection of Transfer RNA Genes in Genomic Sequence. Nucleic Acids Res 25:955–964.

Magrane, M., and U. Consortium. 2011. UniProt Knowledgebase: a hub of integrated protein data. Database 2011:bar009–bar009.

Mair, W., I. Morantte, A. P. C. Rodrigues, G. Manning, M. Montminy, R. J. Shaw, and A. Dillin. 2011. Lifespan extension induced by AMPK and calcineurin is mediated by CRTC-1 and CREB. Nature 470.

Martin, M. 2011. Cutadapt removes adapter sequences from high-throughput sequencing reads. EMBnet J 17.

Martins, R., G. J. Lithgow, and W. Link. 2016. Long live FOXO: Unraveling the role of FOXO proteins in aging and longevity.

Murshid, A., T. Eguchi, and S. K. Calderwood. 2013. Stress proteins in aging and life span. International Journal of Hyperthermia 29.

Nakae, J., M. Oki, and Y. Cao. 2008. The FoxO transcription factors and metabolic regulation.

Peng, S. L. 2008. Foxo in the immune system.

Pertea, M., D. Kim, G. M. Pertea, J. T. Leek, and S. L. Salzberg. 2016. Transcript-level expression analysis of RNA-seq experiments with HISAT, StringTie and Ballgown. Nat Protoc 11.

Pertea, M., G. M. Pertea, C. M. Antonescu, T. C. Chang, J. T. Mendell, and S. L. Salzberg. 2015. StringTie enables improved reconstruction of a transcriptome from RNA-seq reads. Nat Biotechnol 33.

Petersen, T. N., S. Brunak, G. von Heijne, and H. Nielsen. 2011. SignalP 4.0: Discriminating signal peptides from transmembrane regions.

Powell, S., D. Szklarczyk, K. Trachana, A. Roth, M. Kuhn, J. Muller, R. Arnold, T. Rattei, I. Letunic, T. Doerks, L. J. Jensen, C. von Mering, and P. Bork. 2012. eggNOG v3.0: Orthologous groups covering 1133 organisms at 41 different taxonomic ranges. Nucleic Acids Res 40.

Remolina, S. C., P. L. Chang, J. Leips, S. v. Nuzhdin, and K. A. Hughes. 2012. Genomic Basis Of Aging And Life-History Evolution In Drosophila Melanogaster. Evolution (N Y) 66.

Ruderman, N. B., D. Carling, M. Prentki, and J. M. Cacicedo. 2013. AMPK, insulin resistance, and the metabolic syndrome. Journal of Clinical Investigation 123.

Salminen, A., and K. Kaarniranta. 2012. AMP-activated protein kinase (AMPK) controls the aging process via an integrated signaling network.

Salminen, A., K. Kaarniranta, and A. Kauppinen. 2016. Age-related changes in AMPK activation: Role for AMPK phosphatases and inhibitory phosphorylation by upstream signaling pathways.

Simão, F. A., R. M. Waterhouse, P. Ioannidis, E. v. Kriventseva, and E. M. Zdobnov. 2015. BUSCO: Assessing genome assembly and annotation completeness with single-copy orthologs. Bioinformatics 31.

Smit, A., and Hubley R. 2010. RepeatModeler Open-1.0.

Smit, A., R. Hubley, and P. Green. 2010. RepeatMasker Open.

Stanke, M., O. Keller, I. Gunduz, A. Hayes, S. Waack, and B. Morgenstern. 2006. AUGUSTUS: ab initio prediction of alternative transcripts. Nucleic Acids Res 34:W435–W439.

Tricoire, H., V. Battisti, S. Trannoy, C. Lasbleiz, A. M. Pret, and V. Monnier. 2009. The steroid hormone receptor EcR finely modulates Drosophila lifespan during adulthood in a sex-specific manner. Mech Ageing Dev 130.

Ulgherait, M., A. Rana, M. Rera, J. Graniel, and D. W. Walker. 2014. AMPK modulates tissue and organismal aging in a non-cell-autonomous manner. Cell Rep 8.

Yandell, M. 2006. Comparative Genomics Library — RepeatRunner.

Yang, J., and J. Tower. 2009. Expression of hsp22 and hsp70 transgenes is partially predictive of Drosophila survival under normal and stress conditions. Journals of Gerontology - Series A Biological Sciences and Medical Sciences 64.

Zdobnov, E. M., and R. Apweiler. 2001. InterProScan - An integration platform for the signature-recognition methods in InterPro. Bioinformatics 17.
